# Influence of Alternative Protein Sources on the Physicochemical Properties and Sensory Evaluation of a Buffalo Whey‐Based Cocoa Beverage

**DOI:** 10.1111/1750-3841.70999

**Published:** 2026-03-29

**Authors:** Bruna Samara dos Santos Rekowsky, Katherine Gutiérrez‐Álzate, Joseane Cardoso Gomes de Alencar, Bruno Nicolau Paulino, Marion Pereira da Costa

**Affiliations:** ^1^ Program in Animal Science in the Tropics (PPGCAT) Federal University of Bahia Salvador Brazil; ^2^ Program in Food Science, Faculty of Pharmacy Federal University of Bahia (UFBA) Salvador Brazil; ^3^ Department of Bromatological Analysis, Faculty of Pharmacy Federal University of Bahia (UFBA) Salvador Brazil; ^4^ Laboratory of Inspection and Technology of Milk and Dairy Products Federal University of Bahia (UFBA) Salvador Brazil

## Abstract

This study aimed to develop high‐protein and prebiotic buffalo whey‐based cocoa beverages supplemented with calcium caseinate, egg albumin, and rice protein and evaluate their composition, stability, and sensory characteristics. Four formulations with inulin (2.5%) and different protein sources (5.0%) were prepared: BWB – buffalo whey beverage; BCC – beverage with calcium caseinate; BPR – beverage with protein rice; and BEA – beverage with egg albumin. All protein‐enriched beverages contained 7.05–7.53% protein and were classified as high‐protein products. No pathogenic microorganisms were detected, although mesophilic counts reached 6.26–7.12 log CFU/mL after 10 days, which coincided with pH reductions after 15 days (5.95–6.06 in BWB and BPR vs. 6.39–6.83 in BCC and BEA). Viscosity was influenced by protein type, with BWB showing the lowest values (333.5–409.1 mPa·s), BCC the highest values (827.4–1094.7 mPa·s), and BPR/BEA intermediate values (572.7–622.4 mPa·s), comparable to those of smooth samples. As chocolate is a dark chocolate drink, all the treatments were pointed to as having a sweet taste “moderately less than ideal.” BWB, BCC, and BEA achieved acceptable overall liking (“liked it somewhat”), with purchase intentions classified as “undecided” or “probably would buy”. In contrast, BPR was rated “slightly displeased,” primarily due to sandy/grainy mouth feel linked to rice protein solubility. Overall, beverages represent a sustainable and functional alternative and can be easily optimized to improve sweetness and texture, aligning sensory expectations with current trends for functional dairy‐based drinks.

## 1 Introduction

Whey has emerged as a representative byproduct of the dairy industry, accounting for approximately 90% of the milk volume used worldwide for cheese production (Montero‐Zamora et al. 2020; Salgado et al. 2023). Despite its high nutritional value, improper whey disposal can have serious environmental consequences (Magalhães et al. 2011; Silva et al. 2020). The use of milk whey in the development of functional products promotes the advancement of more resilient and sustainable food systems, aligning with the Sustainable Development Goals (SDGs) proposed by the United Nations (Brazil 2018; UN 2015) by supporting alternatives for the full use of agro‐industrial resources, the development of safe and nutritious foods with higher nutritional value and lower environmental impact, while simultaneously strengthening the income of small producers and promoting circular bio economy practices.

Buffalo whey offers a promising alternative for sustainable reuse in dairy systems, providing both nutritional and functional benefits. It contains a well‐balanced profile of essential and nonessential amino acids (Rafiq et al., 2016) and, in vivo studies demonstrate a less intense immune response when compared to cow's milk proteins (Kapila, Kavadi, Kapila, 2013) and has demonstrated anticarcinogenic potential properties (Cacciola et al., 2022). The low total solids content in whey, particularly in fat (0.14% to 0.22%) and protein (1.06% to 1.28%), which is primarily retained in the cheese curd (Di Paolo et al., 2025), presents technological limitations for developing products, such as watery consistency and poor texture formation due to low solids content. Proteins such as B‐Lg and K‐CN, along with calcium concentration, are considered essential for gel formation and the consequent increase in viscosity in dairy products (Ali et al., 2023; Sobti et al. 2020). In addition to proteins, various additives, such as gums, modified starch, and inulin, have been explored to enhance viscosity by increasing the total solids content as a fat replacer (Ba et al. 2025). These strategies can increase viscosity, firmness, and the gel's water‐holding capacity, as well as enhance texture and sensory properties (Sobti et al. 2020). According to Torres et al. (2018), the use of different levels of whey protein micro particles (4.25% and 5.0%) significantly increased the viscosity of skimmed yogurt, for example. Despite the increasing availability of functional ingredients, few studies have addressed the formulation and characterization of multifunctional beverages based on buffalo whey using combinations of animal and plant ingredients with added dietary fiber (Salgado et al. 2023).

Recent studies have explored alternative protein sources to replace or complement meat proteins, including plant‐based ingredients (e.g., cereals, legumes), animal‐derived proteins (e.g., eggs, milk), and byproducts such as edible insects and whey proteins (Otero et al. 2022; Wang et al. 2019). Most protein supplements are based on whey proteins, caseins, eggs, and soy proteins, which are reconstituted with water or milk (Lotfian et al. 2019). These proteins are widely used in high‐protein beverages and nutritional supplements to help meet the recommended daily intake of 0.8–1.0 g per kg of body weight (Huecker et al. 2019; Otero et al. 2022). Blending multiple protein sources has synergistic effects on digestibility, amino acid balance, and functional performance (He et al. 2020; Wang et al. 2018).

Among these sources, calcium caseinate is widely used due to its high solubility and favorable emulsifying and water‐binding properties (Singh and Ye 2019), in addition to contributing to texture stability and antioxidant activity in buffalo dairy products (Atallah et al. 2021). Egg albumin, a low‐cost protein source, is commonly used to increase satiety, contribute to weight control and muscle health, and reduce malnutrition in children (Puglisi and Fernandez 2022). Rice protein, a neglected sustainable byproduct of agricultural processing, has gained attention because of its high digestibility and amino acid composition (Wang et al. 2015, Wang et al. 2018). It balances nutritional value and meets protein intake needs with hypoallergic characteristics but also improves emulsifying properties, increases stability, and extends shelf‐ life (Jayaprakash et al. 2022; Yang et al. 2023). Some studies already bring proposals that can increase the solubilization of rice protein by up to 90%, such as its combination with milk proteins and calcium in alkaline solutions, followed by their neutralization (Wang et al. 2019; Wang et al. 2018), reinforcing the interest in the use of this protein source and expanding its applications in functional food formulations.

Fibers contribute to gastrointestinal health, modulate the glycemic response, and improve the rheological and sensory properties of liquid foods (Kamel et al. 2021; Teferra 2021). In whey‐based matrices, fiber incorporation also improves viscosity, consistency, firmness, and physical stability while enhancing the functional profile of the final product (Guimarães et al. 2018; López‐Castejón et al. 2019; Rosa et al. 2021). The use of calcium caseinate combined with inulin in buffalo whey dairy drinks with vegetable flours with antioxidant properties has already been evaluated (Salgado et al. 2023), providing relevant data about limitations in sensory acceptance due to factors such as texture, mouth feel, and taste.  There is still limited scientific evidence on the combined use of animal‐ and plant‐based proteins in buffalo whey matrices, particularly regarding their technological performance, nutritional quality, and consumer acceptance, pointing to an opportunity to provide systematic studies that address these aspects to support the development of innovative, stable, and sensorially acceptable functional beverages.

According to recent research, the main focuses are the protein characterization of liquid or concentrated buffalo whey, and the investigation of the presence of bioactive compounds such as peptides with health‐beneficial properties (Alfano et al. 2021; Juthi et al. 2025). Regarding the use of buffalo whey in product development, cheeses such as ricotta cheese and symbiotic Petit Suisse cheese are being studied for process optimization and the addition of functional ingredients (Morais et al. 2023; Rashid et al. 2023). As for whey‐based beverages, they are still little studied, and only the work by Salgado and collaborators (2023) proposed the use of buffalo milk whey in its liquid form, also focusing on the use of functional ingredients such as plant flours with antioxidant properties. Thus, to the best of the authors' knowledge, the present work was the first to explore the possibility of developing ready‐to‐drink beverages with a high protein content and prebiotics, made with liquid buffalo whey and using different protein sources as a factor for sensory diversification. In this context, this work enables new ways of valorizing buffalo milk by‐products and more sustainable production systems by reducing whey disposal.

We propose that buffalo milk whey can serve as a viable and sustainable base for the development of protein‐ and prebiotic‐rich beverages, with physicochemical and sensory properties that can be adjusted according to the protein source used. We hypothesize that incorporating alternative protein sources (calcium caseinate, egg albumin, and rice protein) into a beverage based on liquid buffalo milk whey can significantly enhance viscosity, pH stability, and sensory acceptance of non‐fermented, pasteurized beverages. Therefore, this study aimed to develop and characterize high‐protein buffalo whey‐based cocoa beverages supplemented with calcium caseinate, egg albumin, or rice protein. The formulations were evaluated for their proximal composition, sensory properties, color, viscosity, pH, and microbiological stability during refrigerated storage.

## 2 Materials and Methods

### 2.1 Technological Process

The buffalo whey was obtained using the ripened cheese production methodology (Rekowsky et al. 2022) and filtered and characterized using an ultrasonic milk analyzer (Lactoscan Ultrasonic Milk Analyzer Standard; Parana, Brazil). The powdered protein sources with natural flavor were obtained at specialized stores selling supplements, where the calcium caseinate contained 90% protein in its composition (Growth Supplements), rice protein with 80% (Growth Supplements), and egg albumin with 80% protein (Nutraovos). Buffalo whey‐based beverages were prepared using a standardized process following good manufacturing practices adapted from (Salgado et al. 2023).

First, the buffalo whey was pasteurized at 72°C for 15 s and kept frozen until the day the products were prepared. All the dry ingredients, protein sources (calcium caseinate, rice protein isolate, or albumin), cocoa powder, inulin, sucrose, and xanthan gum were weighed and dry mixed. All formulations included cocoa powder (7.50%), inulin (2.25%), sucrose (4.00%), and xanthan gum (0.30%) adapted from (Salgado et al. 2023). Buffalo whey served as the base ingredient, with the volume adjusted to accommodate the added dry ingredients. A total of four treatments were performed: (1) no protein addition (BWB), 5.00% calcium caseinate (BCC), 5.00% rice protein isolate (BPR), and 5.00% albumin (BEA).

Imediatly after defrosting, the whey was heated to 45°C to facilitate the homogenization of the ingredients. The dry mixture was gradually incorporated into preheated (45°C) whey under continuous stirring to ensure homogeneity. The mixture was then homogenized via a food processor (Philips Walita RI7630, 600 W, Philips Do Brasil Ltda, São Paulo, Brazil). All containers and utensils used in processing were previously cleaned and sanitized with food‐grade sanitizing agents, following standard hygienic procedures for dairy processing, in order to minimize post‐processing contamination. The beverages were cooled to 4°C and stored at a refrigeration temperature (4 ± 1°C) for 20 days. At 5‐day intervals, samples were taken to analyze the pH, color, viscosity, and microbiological parameters. This process was repeated for each formulation, ensuring consistency across all the treatments.

### 2.2 Physicochemical characterization

Buffalo milk and whey were analyzed in triplicate using Lactoscan milk analyzers (Lactoscan Ultrasonic Milk Analyzer Standard; Parana, Brazil) to measure the total solids, fat, nonfat (SNF), protein, lactose, ash, and pH values directly.

The buffalo beverages were analyzed for the fat content by the Gerber method, protein by the Kjeldahl method using a conversion factor of 6·38, moisture and total solids contents by oven drying (AOAC 2016). The pH was determined with a pH meter (Model DM‐220; Digimed, São Paulo, Brazil) at 25°C. For all the analyses, three measurements of each aliquot were made to obtain an average value.

Color measurement was performed by using a colorimeter (Konica Minolta CR‐400 Series, Japan) to determine the whiteness (L*), red/greenness (a*), and yellow/blueness (b*) values of the buffalo beverage samples. Before the measurements, the instrument was calibrated with its white reference tile, and three readings were taken from the surface of each sample (Gutierrez‐Álzate et al. 2025). Viscosity measurements were taken at 20°C with a rotational viscometer (Model ViscoQC 300; Anton Paar, Austria) operated at 100 rpm (spindles R4 and R3). Each result was recorded in mPa.s after 10 seconds of rotation, and the average value of three measurements was taken (Akgun et al. 2016).

### 2.3 Microbiological parameters

The mesophilic bacteria were determined on Plate Agar Count (Merck, Darmstadt, Germany) at 37°C for 48 h during storage (0, 5, 10, 15, and 20 days). Yeast and molds were enumerated using DRBC Agar (Dichloran Rose Bengal Chloramphenicol—Merck, Darmstadt, Germany) and incubated at 25°C for 5 to 7 days (APHA 2015). At the same time, the total and thermo tolerant coliform bacteria were determined by the most likely most probable number (MPN) method, which uses lauryl sulfate tryptose broth (HiMedia, Kennett Square, Pennsylvania) as a selective enrichment medium, where positive tubes are inoculated into selective EC broths (HiMedia, Kennett Square, Pennsylvania) of thermo tolerant coliforms such as *E. coli* and bright green broth (Merck, Darmstadt, Germany) for the detection of total coliforms (Queiroz et al. 2025). The microorganisms were enumerated in triplicate.

### 2.4 Sensory analysis

All sensory evaluations were reviewed and approved by the Ethics and Research Committee of UFBA under protocol CAAE 60414022.7.0000.5531. Written informed consent was signed by the participants during the food‐tasting sessions. Individuals with lactose intolerance, allergies to milk and its derivatives, or other ingredients were excluded from this research.

Consumers were randomly recruited based on their interest and willingness to participate in the study conducted at the School of Veterinary Medicine of the Federal University of Bahia, Brazil. There were 100 untrained volunteer participants (73 women and 27 men) aged 18–75 years. Sensory tests were performed in a controlled environment with white lighting and maintained at a temperature between 22 and 24°C with adequate air circulation. The samples were coded with 3‐digit random codes in sequential monadic order following a balanced complete block design and were placed in 50 mL plastic cups and chilled to 4 ± 2°C, and 20 mL of each sample was provided to the assessors. To cleanse the palates of the samples, assessors were provided with salt biscuits and filtered water at room temperature (25°C).

#### 2.4.1 Acceptability and Purchase Intention

In the acceptability test, the panelist was asked to evaluate each sample's appearance, color, odor, flavor, consistency, viscosity, and overall impression using a 9‐point hedonic scale (from 1 = intensely dislike to 9 = extremely like). In contrast, purchase intention was evaluated via a 5‐point scale (1 = certainly would not buy, 5 = certainly would buy) (Salgado et al. 2023).

#### 2.4.2 Check‐All‐That‐Apply (CATA)

The participants received a list of 18 terms in the CATA format. Each participant was asked to select all the terms that described the sensory characteristics identified in each treatment. The expression‐ and attribute‐related terms included taste and flavor (whey flavor, chocolate flavor, strange taste, “resemble egg or rice,” “easy to drink,” and “make you thirsty”), aroma (cocoa aroma, whey aroma, sweet aroma, rice or egg aroma), appearance (intense brown color, light brown color, homogeneous appearance, “looks appetizing,” “looks healthy,” and “looks unhealthy”), and texture (sandiness, liquid consistency, thick/creamy).

#### 2.4.3 Just‐About‐Right (JAR)

The jJAR sensory test evaluated the optimal intensity of sensory attributes in the buffalo whey‐based beverage with high protein and fiber content. The consumers evaluated attributes such as aroma (cocoa, whey milk), taste (sweet, sour), flavor (milk chocolate, dark chocolate), color (dark brown, light brown), and texture (sandiness, consistency, firmness, viscosity, mouth feel) via a JAR scale with five points (1 = not enough, 3 = ideal, and 5 = too much), as described previously (Salgado et al. 2023).

### 2.5 Statistical analysis

The experiment followed a completely randomized design with a factorial arrangement of treatments (four formulations: BWB, BCC, BPR, and BEA) and, when applicable, storage time (0, 5, 10, 15, and 20 days). All physicochemical, instrumental, and microbiological analyses were performed in experimental duplicate and analytical triplicate, and results were expressed as the mean ± standard deviation.

The parameters of formulations during storage time on composition (moisture, total solids, protein, and fat) were analyzed at the initial and end of the storage period (0 and 20 days) while the effects of formulation and storage time on pH, color parameters (L*, a*, b*), apparent viscosity were evaluated during selected points of storage (0, 5, 10, 15, and 20 days) using two‐way ANOVA, followed by Tukey's post hoc test (*p* < 0.05). Microbiological counts for mesophilic bacteria were log‐transformed (log CFU/mL) and also subjected to analysis of variance (ANOVA) followed by Tukey's post hoc test.

The acceptance and purchase intention were analyzed by analysis of variance (ANOVA) followed by Tukey's post hoc test, with *p <* 0.05 (95% confidence intervals). Penalty analysis was applied to JAR data to identify the impact of non‐ideal attribute intensities (“too little” or “too much”) on overall liking, calculating the mean drop in acceptance scores and the proportion of consumers penalizing each attribute. For CATA analysis, each descriptor's frequency was calculated as the number of consumers who used it to describe each sample, and Cochran's Q test (*p <* 0.05) was used to determine differences between the samples for each descriptor. All statistical analyses were performed in the XLSTAT program (Version 2013.2.03; Addinsoft, Paris, France).

## 3 Results and Discussion

### 3.1 Milk and Whey Characterization

Approximately 47.38 ± 0.40% of the total solids present in buffalo milk were recovered in the whey, mainly lactose, mineral salts, and proteins, respectively. In comparison, 91.84 ± 0.09% of fat was retained in cheese production (Table 1), indicating that buffalo whey milk is a good raw material for developing dairy products with high nutritional and low‐fat values. Whey protein comprises mainly α‐lactalbumin, β‐lactoglobulin, and serum albumin, followed by lactoferrin and lactoperoxidase (Ostertag et al., 2021), and shows the highest amino acid release after digestion, providing a protein source with good digestibility and a good ratio of essential/nonessential amino acids (Almeida et al. 2016).

**TABLE 1:** Proximal compositions of buffalo milk and whey protein (means ± SDs).

Parameters	Proximal composition (%)					
	Fat	Protein	Lactose	Ash	Total solids	pH
Buffalo milk	6.78 ± 0.04	3.39 ± 0.05	5.12 ± 0.08	0.76 ± 0.01	16.10 ± 0.18	6.87 ± 0.02
Buffalo whey*	0.55 ± 0.01	2.61 ± 0.01	3.89 ± 0.02	0.57 ± 0.01	7.63 ± 0.03	6.13 ± 0.03

*Note*: *Buffalo milk whey is obtained from the production of probiotic cheese by enzymatic coagulation.

The recovery of milk constituents in whey is valuable information in the context of industrial efficiency, as protein and fat constituents are lost less during cheese process than lactose is (Sales et al. 2018). Milk protein consists of two principal groups of favorable essential and nonessential amino acid sources. In buffalo milk, the average protein content is 3.87%, with 3.20% corresponding to casein, and 0.68% to whey proteins (Rafiq et al. 2016). The protein content in liquid whey from cows (2.69%) and buffaloes (2.86%) analyzed by us was similar to Salgado et al. (2023) results. When used to produce dairy beverages, formulations from buffalo whey can expand the range of products with different qualities than those traditionally made with cow's milk and reduce their cost (Silva et al. 2020).

### 3.2 Beverage Composition

The control beverage (BWB) had the highest moisture content (80.13–79.52%), which can be attributed to the absence of added protein ingredients (Table 2) and resulted in the lowest (*p <* 0.05) total solids content (19.87–20.48%) throughout the storage period. In the symbiotic buffalo whey drink, higher values were observed for moisture (82.93%), and a lower total solids content (17.08%) was affected mainly by lower amounts of protein (0.43%) and fat (0.25%) (Kumar et al. 2021).

**TABLE 2:** Physicochemical characterization of high protein buffalo whey prebiotic beverages.

Treatment	Storage (days)	BWB	BCC	BPR	BEA
Moisture (%)	0	80.13 ± 0.01^a*^	74.44 ± 0.10^c*^	74.86 ± 0.13^b*^	73.09 ± 0.42^d*^
	20	79.52 ± 0.08^a**^	75.54 ± 0.02^b*^	73.09 ± 0.42^c*^	72.96 ± 0.33^d*^
Total solids (%)	0	19.87 ± 0.01^d*^	25.56 ± 0.11^c*^	26.16 ± 0.14^b*^	26.91 ± 0.49^a*^
	20	20.48 ± 0.09^d**^	24.46 ± 0.03^c*^	25.14 ± 0.15^b*^	27.04 ± 0.37^a*^
Protein (%)	0	2.24 ± 0.03^c*^	7.05 ± 0.12^b*^	7.44 ± 0.05^a*^	7.48 ± 0.02^a*^
	20	2.30 ± 0.05^c**^	6.93 ± 0.14^b*^	7.53 ± 0.04^a*^	7.50 ± 0.06^a*^

Abbreviations: BWB = buffalo whey beverage; BCC = beverage with calcium caseinate; BPR = beverage with protein rice; BEA = beverage with egg albumin.

*Note*: Different lowercase letters on the same line indicate statistically significant differences (*p* < 0.05) between treatments.

Different * values in the same column indicate a statistical difference in parameters (*p* < 0.05) during storage time.

^#^The limits are below what is detectable via the Gerber method.

Compared with the control beverage (2.24–2.30%), all formulations with added protein sources presented significantly greater protein contents (6.93–7.53%; *p* < 0.05). Protein levels remained stable between day 0 and day 20 (*p* > 0.05) in all formulations, demonstrating protein stability during refrigerated storage. The BCC treatment showed statistically compared with both the control and the other protein formulations, whereas no significant differences (*p* > 0.05) were detected between the treatments with isolated rice protein (BPR) and egg albumin (BEA), indicating similar performance. These results are similar to those reported in high‐protein cocoa milk beverages by Helal et al. (2023), who used milk and pasteurized eggs as protein sources and reported values between 3.24% and 7.12% for protein in 21.32% to 22.19% of total solids in the beverage. Using milk protein isolated with oat flour can generate increased protein content drink with approximately 5.16% to 6.47% protein (Vasquez‐Orejarena et al. 2018). The protein content in commercial products varies due to the use of raw materials such as soy milk products (2.88% to 3.16%), rice milk (0.28%), almond milk (0.31% to 2.08%), coconut milk (0.59% to 2.0%) (Chalupa‐Krebzdak et al. 2018), or 3.42% protein in amaranth beverages (Manassero et al. 2020), indicating that few plant‐based alternatives can significantly contribute to the daily protein intake without the addition of specific ingredients.

On the other hand, the lower protein concentration in BCC than those in high protein formulations may be attributed to the physicochemical behavior of caseinates in aqueous systems. Caseinates exhibit limited solubility under mildly acidic or near‐neutral pH conditions, which can promote protein aggregation or precipitation, thereby reducing the measurable protein content in solution (Holt and Carver 2024). Casein molecules readily interact with calcium, phosphate, and anionic hydrocolloids, forming insoluble complexes that further decrease protein solubility. These factors can explain the lower apparent protein levels detected in the BCC formulation, despite equivalent protein addition across treatments (Holt and Carver, 2024).

In the population aged 19–50 years, the reference daily intake (RDI) of protein is 0.80 g/kg/d for protein (Chalupa‐Krebzdak et al. 2018; Huecker et al. 2019), which, for a body weight of 60 kg, approximately 50 g/day, is the ideal amount of protein intake. Considering a common commercially available “ready‐to‐drink” beverage with a serving size of 240 mL (8fl‐oz), the BCC, BPR, and BEA treatments provide approximately 16.0 to 18.0 g of protein, which guarantees that at least 32.0% to 36.0% of the RDI is an increased protein content beverage (>20% RDI). In contrast, the BWB treatment provided approximately 5.4 g of proteinper serving. The amount of protein per serving (g/240 mL serving) in “ready‐to‐drink” protein beverages can range from 9.0 to 23.8 g per serving (Liu et al. 2021). Whey‐based beverages are an excellent way to reuse the liquid whey produced from cheese manufacturing, and this type of “ready‐to‐drink” beverage is widespread and popular among consumers, being a profitable and sustainable business (Guimarães et al. 2018).

All four formulations were prepared with 2.25% inulin, a prebiotic fiber that adds functional value to the product. Considering a standard commercially available “ready‐to‐drink” beverage, a serving size of 240 mL (8fl‐oz) provides approximately 5.40 g of dietary fiber. The RDI of dietary fiber for adults (aged ≥ 19) is 25g/day, which is the ideal amount of fiber intake (Brazil, 2020). Inulin is considered a prebiotic dietary fiber that selectively stimulates the growth and viability of beneficial health groups, such as Lactobacilli Bifidobacteria microorganisms (Kamel et al. 2021; Parhi et al. 2021), but when consumed in excess (over a daily intake of 14–18 g), it can promote intolerance effects, such as increased flatulence, abdominal cramping, and bloating (Teferra, 2021).

All beverages can be characterized by low‐fat content (<0.5%), a source of dietary fiber, a clean label composition, sustainable production and a novel approach to the valorization of a byproduct in the dairy industry, making it an appealing base for the development of functional, low‐fat beverages. Moreover, since buffalo whey‐based cocoa beverages are not yet available on the market, even the control treatment holds potential as an innovative, nutritious, and sustainable alternative for future product development.

### 3.3 Microbiology

**FIGURE 1:**
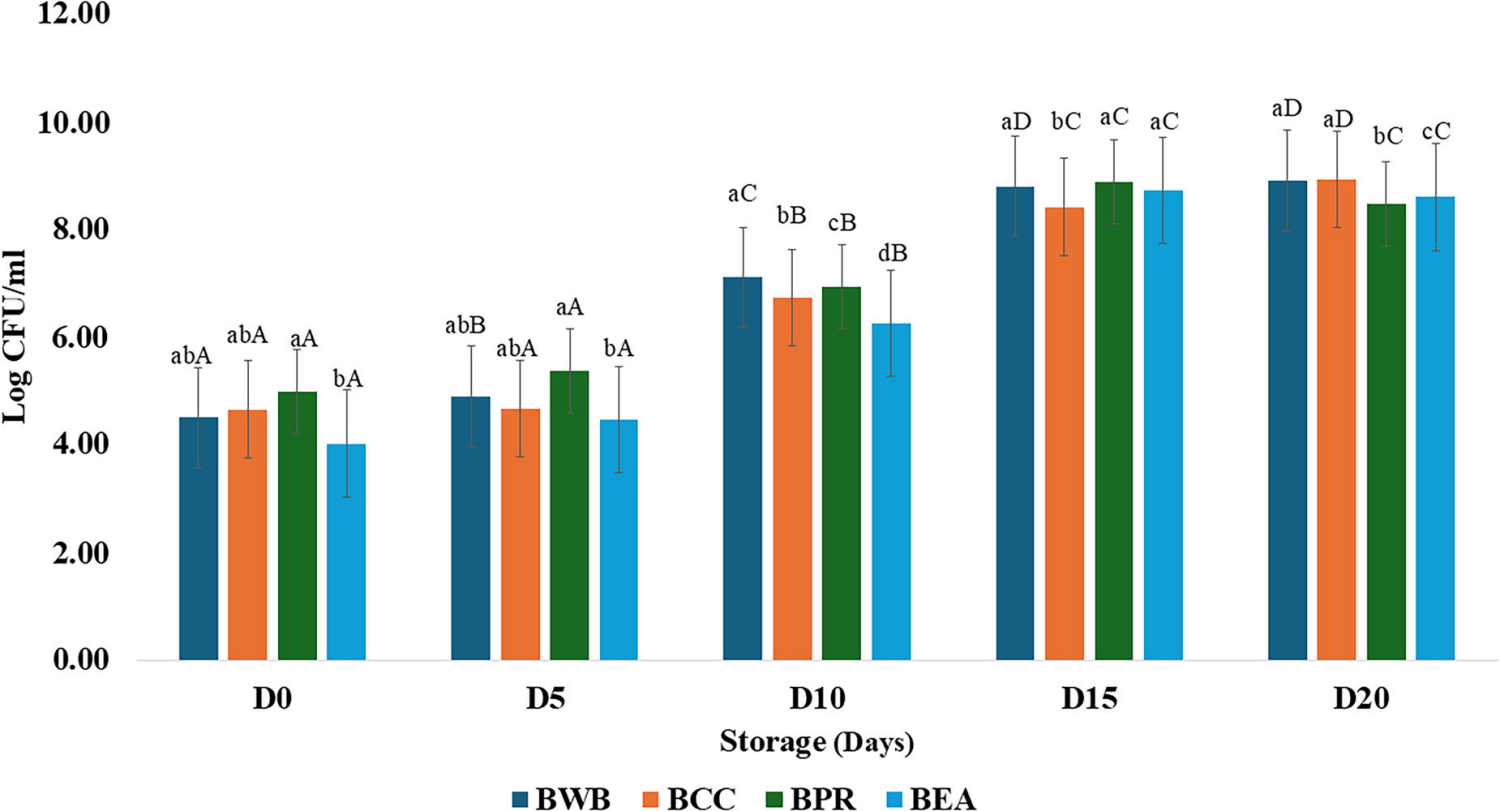
Average viable count of mesophilic bacteria in buffalo whey‐based cocoa beverages after 0, 5, 10, 15, and 20 days of storage at 4 ± 1°C. Different lowercase letters indicate significant differences (*p* < 0.05) among treatments at the same storage time. Different uppercase letters indicate significant differences (*p* < 0.05) over storage time within the same treatment.

To evaluate the microbiological quality and hygienic conditions of the whey‐based cocoa beverage samples, total coliforms and *E. coli* were quantified. Total coliforms and *E. coli* were not detected in any samples during refrigerated storage. The mesophilic counts (Figure 1) on the first day of storage ranged from 4.03 to 5.00 log CFU/mL in the treatments. According to Lotfian et al. (2019), pasteurized high‐protein cocoa beverage products start storage at approximately 3.0 to 4.0 log CFU/mL, which demonstrates that the thermal process of sterilization is more effective than pasteurization in reducing the number of bacteria in this type of product.

A continuous increase in the total bacterial count was observed, and after 10 days of storage, the BWB treatment resulted in high counts of mesophiles (7.12 log CFU/mL), whereas the other treatments resulted in counts between 6.26 and 6.94 log CFU/mL. The bacterial counts in the treatments ranged from 8.48 to 8.94 log CFU/mL during the final storage period. Similar results were observed in cocoa and whey protein‐enriched functional dairy drinks, which significantly increased from 0–18 days of storage (Gupta et al. 2017). It can be speculated that prebiotic fibers such as inulin might form a gel matrix around bacterial cells, protecting these microorganisms from injury during storage, resulting in a 4–5% increase in viability with inulin supplementation and lactose presence (Parhi et al. 2021). The total plate count increases proportionally as the level of whey increases since this can be considered a greater availability of nutrients (lactose sugars and proteins) favorable for microorganism growth (Panghal et al. 2017).

Thus, considering whey obtained from cultured cheese, along with most starter cultures, and probiotic cells, the highest viable cell counts can be explained by the exponential growth that occurs during the technological process of fermented products. Thermal sterilization may be more suitable for ensuring the quality and shelf‐life of whey‐based products. However, considering the increased relevance of nonfermented probiotic products, which are considered products containing probiotics without fermentation (Jang et al. 2024), this viability can indicate that the formulation is a suitable matrix for probiotic beverages since, in nonfermented milk, after 14 days of storage (4°C), probiotic lactic acid bacteria can have viable counts between 8.40 and 8.50 log CFU/mL without significantly affecting the pH of beverages (Jang et al. 2022).

### 3.4 pH values

**FIGURE 2:**
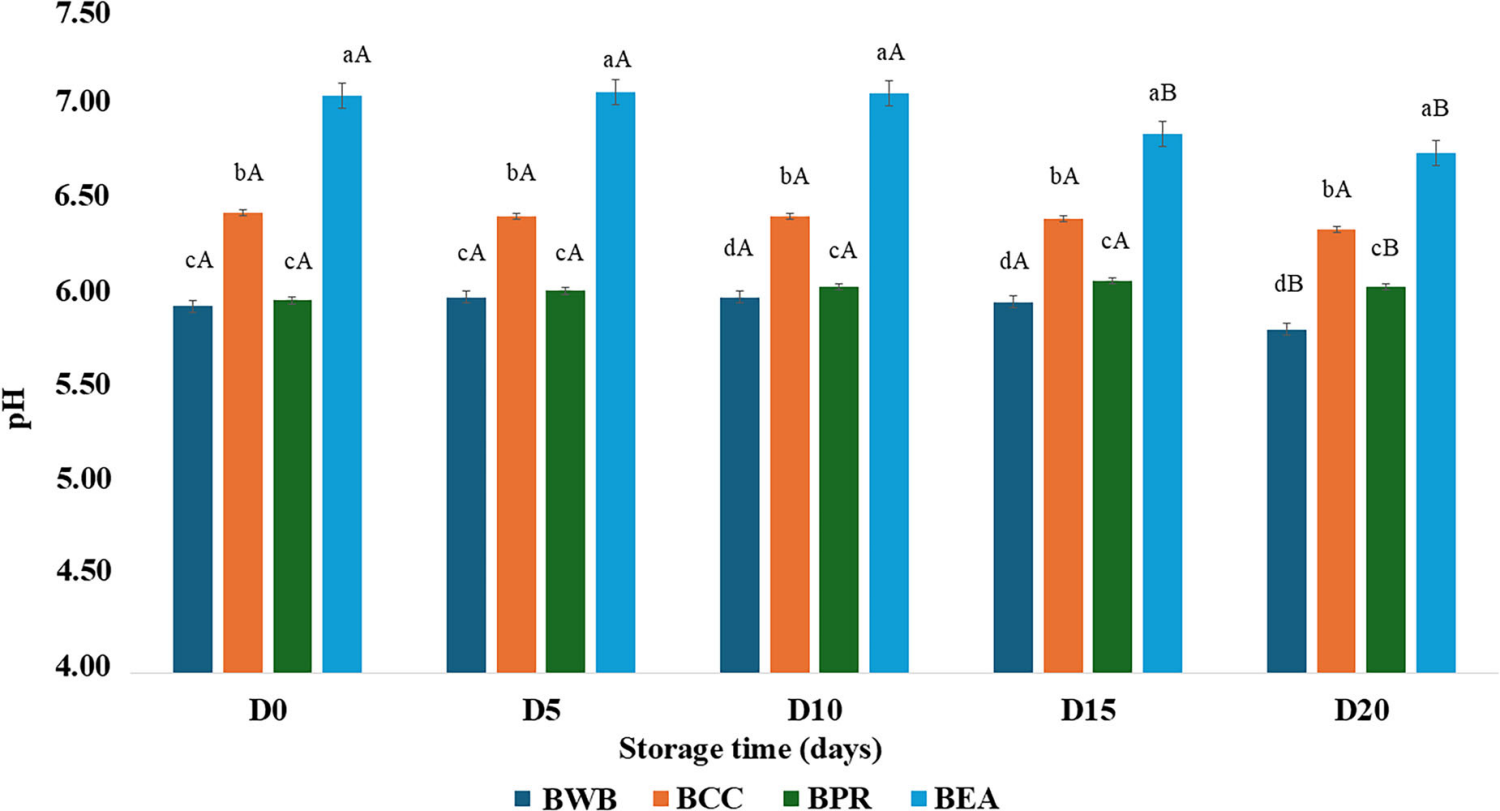
pH of buffalo whey‐based cocoa beverage formulations during 20 days of refrigerated storage at 4 ± 1°C. BWB = buffalo whey beverage (control); BCC = beverage with calcium caseinate; BPR = isolated protein rice; and BEA = beverage with egg albumin. Different lowercase letters indicate significant differences (*p* < 0.05) among treatments at the same storage time. Different uppercase letters indicate significant differences (*p* < 0.05) over storage time within the same treatment.

Overall, the egg albumin formulation (BEA) had significantly greater pH values (*p <* 0.05) throughout the storage period, ranging from 7.04 on day 0 to 6.74 on day 20 (Figure 2). Although it maintained the highest pH among the formulations, a significant decrease (*p <* 0.05) was observed from day 15, indicating the beginning of acidification. In dairy beverages, the addition of white egg powder or pasteurized eggs significantly increased the pH of enriched samples, indicating the natural alkaline properties of this ingredient (Helal et al. 2023; Lotfian et al. 2019).

The control (BWB) and the rice protein beverage (BPR) did not differ (*p >* 0.05) from each other during the early days of storage, with pH values ranging from approximately 5.98–6.01 on day 5. However, after day 10, BWB began to acidify more intensely (*p <* 0.05) than the other treatments did, reaching a pH of 5.81 by day 20, the lowest among all the treatments. In contrast, BPR maintained stable pH values until day 15, after which a slight decline was observed. The pH of the buffalo whey used to produce beverages is close to 6.13. Both the control (BWB) and the BPR samples started storage at pH 5.93 and 5.96, respectively. It can be assumed that the amount of cocoa powder did not significantly influence this parameter, but increased total solids, such as protein and inulin, and can positively impact the stability of the products. The natural cocoa powder has a pH close to 6.0–7.2 and, along with other ingredients, pH changes over the storage period can affect the color parameter, since the higher the pH, the darker the beverage appears (Barišić et al. 2023; Juvinal et al. 2023).

Notably, the calcium caseinate formulation (BCC) was the only treatment that did not undergo significant acidification (*p* > 0.05) during the storage period, with pH values ranging narrowly from 6.42 to 6.33, indicating excellent physicochemical stability under refrigerated conditions. The pH of calcium caseinate can reach values close to 6.83 (Atallah et al. 2021). The pH of whey‐based drinks can be influenced by several factors, including the type of cheese from which it is derived (acid or enzymatic coagulation) and the characteristics of the added ingredients. The shelf‐life of whey beverages is considered microbiological, physicochemical, and sensory aspects, and, in fermented beverages, can reach 9 to 10 days, with a pH reaching 3.6 (Kumar et al. 2021). In nonfermented whey drinks, the pH can decrease from 5.46 to 4.75 within 28 days but has an adverse effect on the taste of the product after 7 days of storage, which is significantly affected by the characteristics of the ingredients used in the formulation (Zaman et al. 2023).

### 3.5 Apparent viscosity

**FIGURE 3:**
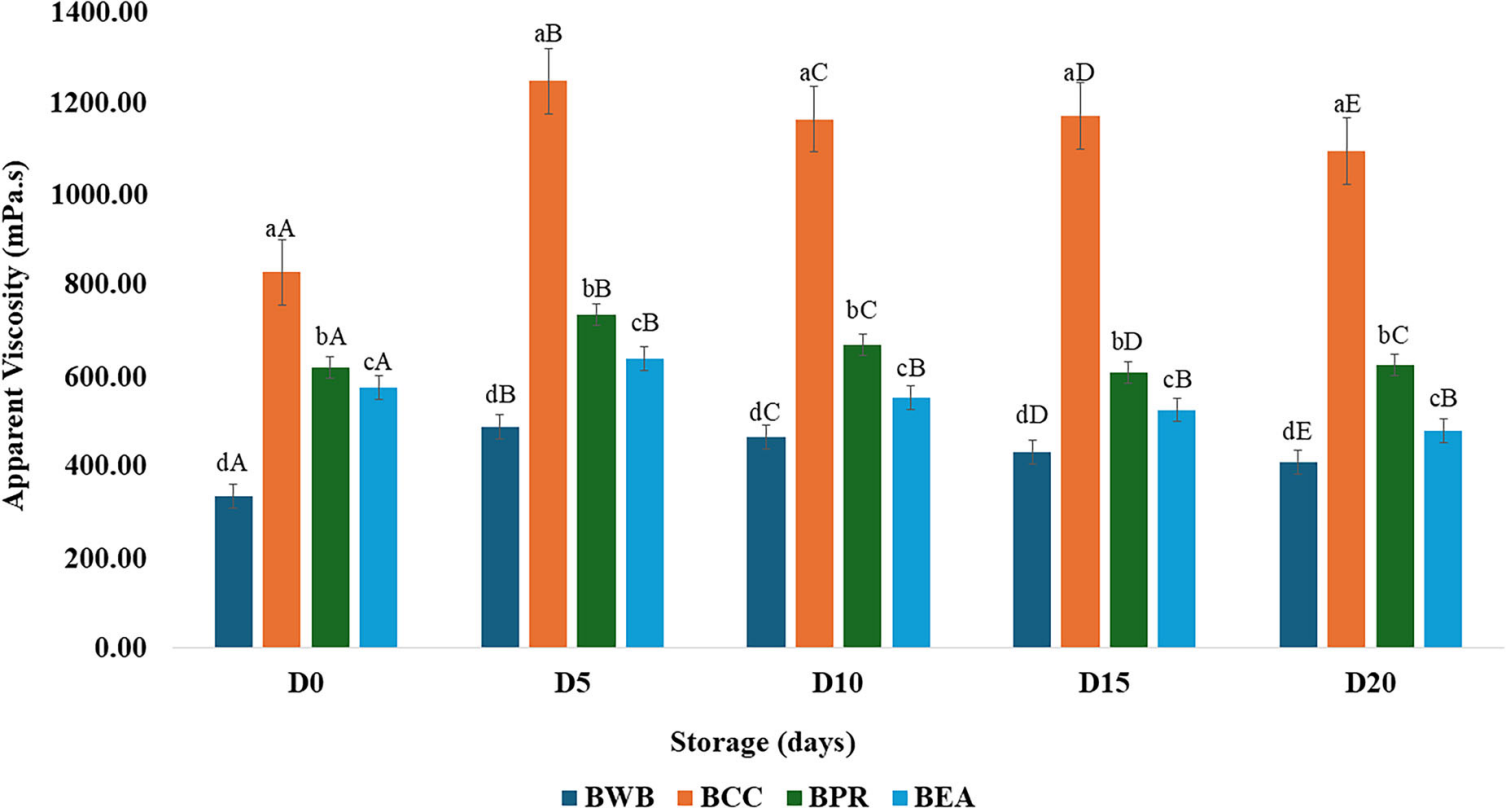
Apparent viscosity in buffalo whey‐based cocoa beverages during 20 days of storage. BWB = buffalo whey beverage; BCC = beverage with calcium caseinate; BPR = beverage with isolated protein rice; and BEA = beverage with egg albumin. Different lowercase letters indicate significant differences (*p* < 0.05) among treatments at the same storage time. Different uppercase letters indicate significant differences (*p* < 0.05) over storage time within the same treatment.

During refrigerated storage, all formulations exhibited distinct rheological behaviors (Figure 3). The BCC sample presented the highest initial viscosity (∼850 mPa·s) (*p <* 0.05), whereas BPR and BEA presented similar (*p* > 0.05) initial viscosities, which reached values between 572.73 and 618.10. Caseinate suspensions are viscoelastic liquids, and for calcium caseinate, the viscosity increases strongly with increasing concentration and decreasing temperature due to attractive interactions mediated by Ca2+, inducing the formation of dense domains of the protein (Thomar et al. 2013) that act as efficient emulsifiers at high or intermediate concentrations in a temperature‐dependent manner, resulting in high values at lower temperatures (Gupta and Ghosh, 2015). BCC may have a greater viscosity and a creamy feel because of the ability of calcium caseinate to form stable structures.

In contrast, the control treatment (BWB), consisting solely of buffalo liquid whey and inulin, exhibited the lowest initial viscosity throughout the storage period (*p* < 0.05), starting at 333.50 mPa·s and reaching a maximum of only 487.47 mPa·s on the 5th day of storage. All the treatments presented an increase in viscosity in the first 5 days of storage, after which the viscosity remained stable until the 20th day of storage. After 72 hours of storage, an increase in viscosity is observed in beverages with whey protein and inulin, indicating a late effect of inulin on the particle size distribution (Guimarães et al. 2018). Heat treatment can naturally induce a redistribution of polypeptides in different aggregates by weakening hydrogen bonds followed by the formation of new bonds and can increase the particle size in the presence of gums (xanthan and gellan gum), forming macrocomplexs in a network similar to that of a gel, increasing viscosity and contributing to the physical stability of the product (Manassero et al. 2020). Rice proteins are excellent ingredients due to their emulsifying, gelling, water‐holding capacity, and oil‐holding capacity properties (Jayaprakash et al. 2022; Yang et al. 2023).

Despite the absence of fruit pulp or ice cream as ingredient, with a naturally higher protein content and no water addition, our buffalo whey‐based formulation could reach an apparent viscosity similar to that of smoothies or milkshakes. However, prebiotic beverages usually present low viscosity (11.0 to 29.2 mPa.s), even when inulin (6%) and gum (0.05%) are added, which is attributed to the use of 35 to 41% water as an ingredient (Guimarães et al. 2018), and when whey buffalo milk, inulin, and pasteurized water whey‐based beverages are used, the viscosity (10.6 mPa.s) and protein content are low (Kumar et al. 2021). A smoothie is a nonalcoholic beverage generally made from a combination of fruits and processed vegetables supplemented with dairy and plant‐based ingredients that are usually related to healthy food and are typically smooth and thick (Panda et al. 2023; Rani et al. 2024). In high‐protein and high‐fiber smoothies with soy protein isolate and pectin, the viscosity varies from 357 to 728 mPa.s (Mehta et al. 2017) or from 718 to 880 mPa.s in smoothies with increased total solids (24.38% to 29.40%) in plant‐based products (Panda et al. 2023).

### 3.6 Color

All beverages were formulated with 7.5% cocoa powder (semi‐sweet chocolate), which contributed significantly to the characteristic brown hue observed across treatments (Table 3) as the values were greater in the dark (L* = 0) than in the white state (L* = 100). Cocoa is a component associated with a characteristic light brown color, and the amount of cocoa has a greater effect on beverage luminosity, which decreases with increasing amounts of cocoa bean 380 (Pérez‐Ramírez et al. 2021). All formulations presented redness (positive values for a*) and yellowness (positive values for b*) characteristics. However, the formulations still exhibited significant differences depending on the protein source. The color of cocoa whey‐based beverages depends on the medium in which the beverages are prepared (Barišić et al. 2023).

**TABLE 3:** Color parameters during 20 days of storage of the buffalo whey‐based cocoa beverage.

Parameters	Storage (Days)	Treatments^#^			
		BWB	BCC	BPR	BEA
L*	0	42.06 ± 0.14^c^	44.78 ± 0.06^b^	46.64 ± 0,01^a^	41.66 ± 0.03^d^
	5	42.55± 0.31^c^	44.77 ± 0.01^b^	46.51 ± 0.01^a^	41.49 ± 0.03^d^
	10	42.77 ± 0.05^c^	44.75 ± 0.11^b^	46.48 ± 0.01^a^	41.55 ± 0.04^d^
	15	42.81 ± 0.03^c^	44.57 ± 0.06^b^	46.09 ± 0.03^a^	41.49 ± 0.04^d^
	20	42.30 ± 0.10^c^	44.28 ± 0.09^b^	45.90 ± 0.03^a^	41.49 ± 0.08^b^
a*	0	6.22 ± 0.02^b^	4.93 ± 0.08^c^	6.82 ± 0.02^a^	4.91 ± 0.03^c^
	5	6.01 ± 0.01^b^	4.87 ± 0.02^c^	6.86 ± 0.01^a^	4.76 ± 0.02^d^
	10	6.08 ± 0.03^b^	4.88 ± 0.02^c^	6.84 ± 0.02^a^	4.79 ± 0.02^d^
	15	5.78 ± 0.02^b^	4.67 ± 0.19^c^	6.87 ± 0.02^a^	4.83 ± 0.04^c^
	20	5.94 ± 0.05^b^	4.80 ± 0.02^d^	6.93 ± 0.01^a^	5.40 ± 0.02^c^
b*	0	6.13 ± 0.02^b^	5.29 ± 0.04^c^	8.54 ± 0.02^a^	4.43 ± 0.04^d^
	5	5.97 ± 0.01^b^	5.28 ± 0.02^c^	8.54 ± 0.16^a^	4.32 ± 0.05^d^
	10	6.13 ± 0.05^b^	5.25 ± 0.06^c^	8.60 ± 0.01^a^	4.27 ± 0.02^d^
	15	5.73 ± 0.02^b^	5.28 ± 0.03^c^	8.62 ± 0.01^a^	4.25 ± 0.05^d^
	20	5.94 ± 0.04^b^	5.06 ± 0.05^c^	8.67 ± 0.04^a^	5.08 ± 0.04^c^

Abbreviations: BWB = buffalo whey beverage; BCC = beverage with calcium caseinate; BPR = beverage with isolated protein rice; BEA = beverage with egg albumin.

Note: L* indicates the brightness of the product ranging from absolute white (value 100) to absolute black (value 0); a* represents the variation from green (–) to red (+); and b* represents the variation from blue (–) to yellow (+).

Different lowercase letters on the same line indicate statistically difference (*p* < 0.05) between treatments.

^#^ No difference was observed in the same column indicating no statistical difference in parameters (*p* > 0.05) during storage time.

The rice protein beverage (BPR) significantly (*p <* 0.05) presented the highest values for the color parameters L*, a*, and b*, indicating a lighter and more saturated reddish‐yellow tone (Table 5). The characteristics of the rice protein powder indicate a light‐colored ingredient (L* = 72.70), with a* value close to 7.79 and prominent yellowness (b* = 40.82), indicating a sandy/beige color characteristic (Ahern et al. 2025). In contrast, the BEA (egg albumin beverage) presented the lowest (*p* < 0.05) L* and b* values throughout storage, resulting in a darker and less yellow appearance. Egg white powder affects color because of the yellowish‐white color, so the greater the amount of albumin used, the greater the degree of yellowness of the product (Romulo and Aurellia 2024).

During the 20‐day refrigerated storage, a slight but non‐significant decrease in L* (*p* > 0.05) was observed in all treatments, particularly in BPR and BCC, which can be attributed to oxidation or interactions with the protein matrix, resulting in mild darkening. The color of cocoa whey‐based beverages depends on the medium in which the beverages are prepared, pH and bioactive components such as polyphenols, mainly proanthocyanidins, and anthocyanins that present relevant antioxidant activity (Barišić et al. 2023) and may have contributed positively to maintaining the visual characteristics of the formulations throughout the storage period. While the a* and b* values remained relatively stable for BPR and BWB, an increase (*p* < 0.05) in these parameters was observed in BEA by day 20. The perception of redness or greenness (a* values) is due to compounds in the product responsible for absorbing light at specific wavelengths with consequent reflectance of green or red light, so positive values indicate redness, and negative values indicate greenness (Cheng et al. 2018). The protein source could affect color stability and perception despite the uniform addition of cocoa powder. BPR demonstrated superior visual consistency and brightness, which could enhance consumer appeal for chocolate‐flavored dairy‐like beverages. Consumers can anticipate other sensory characteristics, such as the taste and flavor of a product, based on its color (Ahern et al. 2025; Juvinal et al. 2023).

### 3.7 Sensory Acceptance

**FIGURE 4:**
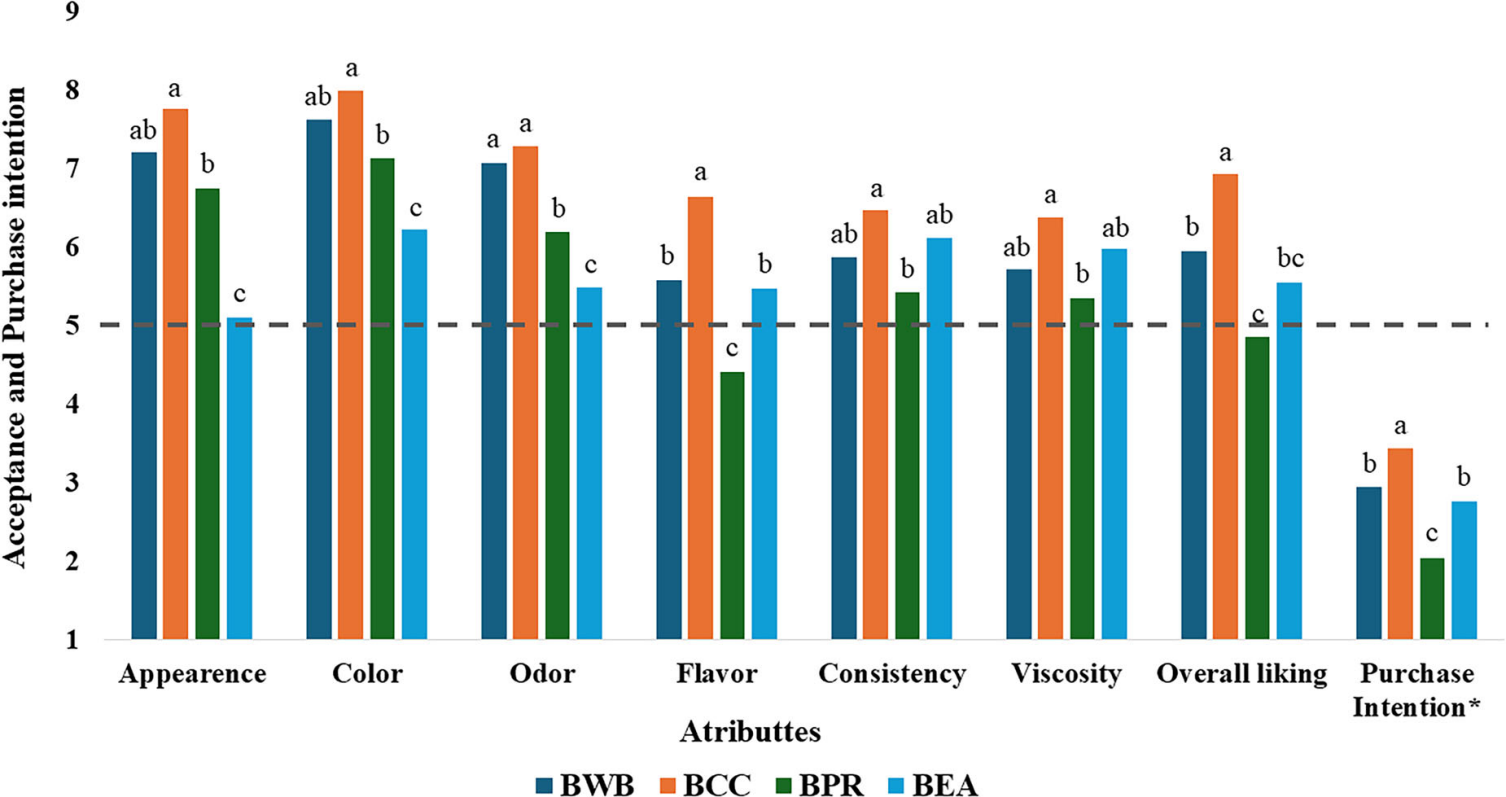
Effect of different protein sources on the sensory acceptance attributes (means ± SDs) of buffalo whey‐based coca beverages. BWB = buffalo whey beverage serves as a reference, being the basic formulation with only buffalo milk whey; BCC = beverage with calcium caseinate; BPR = beverage with isolated protein rice; and BEA = beverage with egg albumin. Sensorial attributes were evaluated on a hedonic scale of 9 points; *purchase Intention was evaluated on a 5‐point scale. Lowercase letters with the same attributes indicate significant differences between treatments (*p* < 0.05).

The results of the sensory attribute acceptance of the high‐protein and high‐fiber buffalo whey‐based cocoa beverage are shown in Figure 4. Except for BPR, all the treatments presented an acceptable overall liking (above 5.0). BCC was significantly well rated (*p <* 0.05), reaching an acceptance score of 6.94 (I moderately liked it) compared with BWB (5.95) and BEA (5.55), which were classified between “I neither liked nor disliked” and “I slightly liked it”.

The appearance, color, and odor of BEA were negatively influenced (*p <* 0.05) by the addition of egg albumin powder since excess foam formation and a more intense color added by a residual egg aroma were observed by some consumers as negative characteristics. After 30 min of storage, the foam coarsening rates of rice protein and caseinate formulations decreased significantly, probably because the composite proteins formed a more compact air–liquid interface layer (Zhang et al. 2024). According to Helal et al. (2023), increasing the proportion of eggs as ingredients can reduce sensory quality in terms of aroma, color, and appearance. However, the use of vanilla as a flavoring agent can be a strategy to improve these characteristics. On the other hand, an intermediate viscosity associated with the mild flavor of egg albumin masked by cocoa powder positively reflected the texture, flavor, and overall acceptance of the product.

Flavor was a significant factor that negatively impacted (*p <* 0.05) the score in beverages supplemented with rice protein (BPR). Despite their wide range of sources and low cost, developing attractive plant protein products remains challenging because of their typically unfavorable sensory perception (He et al. 2020). The texture characteristics (viscosity and consistency) of the different beverages were well assessed. The higher viscosity of BCC was a positive characteristic, as it was noted for its high consistency and creaminess, resembling a dairy dessert. For BPR, despite the homogeneous appearance, a sandy sensation in the mouth feel was observed as a negative aspect. The textural characteristics of dairy products are important parameters because of their important role in consumer acceptance (Akalin et al. 2012). The enrichment of products by enhancing the level of dry matter with caseinates can provide improved texture and nutritional properties, such as increased levels of protein in the product (Remeuf et al. 2003).

Considering purchase intentions, the BCC treatment exhibited the highest potential (*p <* 0.05) for consumer interest, with evaluations indicating a likelihood between “I would probably buy it” and “Might or might not buy” (scores close to 3.5) and similarities in BWB and BEA treatments, indicating a neutral interest from consumers in purchasing these products with a score close to 3.0 (“might or might not buy”). The BPR treatment received the least favorable evaluation (*p <* 0.05), with an apparent inclination to “probably would not buy” (scores around two) by consumers.

The use of CATA to describe the sensory characteristics of products allows an in‐depth analysis of consumer perception and differences between protein sources. This approach is useful for identifying attributes that can guide the development of future products. From all the attributes analyzed (Table 4), whey flavor, sweet aroma, and characteristic expressions such as “makes you thirsty,” “looks healthy,” and “looks unhealthy” did not present a difference (*p >* 0.05) in the perceptions of consumers regarding the treatments.

**TABLE 4:** The consumers indicated the frequency (%) of each term of the CATA for different formulations of buffalo whey‐based cocoa beverages.

Characteristics	BWB (%)	BCC (%)	BPR (%)	BEA (%)	*p‐*Values
Whey flavour	19.00 ± 0.39^a^	11.00 ± 0.31^a^	24.00 ± 0.43^a^	16.00 ± 0.37^a^	0.068
Cocoa flavour	71.00 ± 0.46^a^	80.00 ± 0.40^a^	38.00 ± 0.49^b^	62.00 ± 0.49^a^	<0.0001
Strange taste	15.00 ± 0.36^b^	11.00 ± 0.31^b^	46.00 ± 0.50^a^	23.00 ± 0.42^b^	<0.0001
It resembles egg, rice, or whey	7.00 ± 0.26^b^	6.00 ± 0.24^b^	29.00 ± 0.46^a^	36.00 ± 0.48^a^	<0.0001
Makes thirsty	15.00 ± 0.36^a^	20.00 ± 0.40^a^	21.00 ± 0.41^a^	16.00 ± 0.37^a^	0.480
Easy to drink	54.00 ± 0.50^a^	35.00 ± 0.48^b^	19.00 ± 0.39^c^	34.00 ± 0.48^bc^	<0.0001
Liquid consistency	65.00 ± 0.48^a^	2.00 ± 0.14^c^	8.00 ± 0.27b^c^	19.00 ± 0.39^b^	<0.0001
Thick/Creamy	18.00 ± 0.39^c^	93.00 ± 0.26^a^	46.00 ± 0.50^b^	59.00 ± 0.49^b^	<0.0001
Sandiness	21.00 ± 0.41^b^	25.00 ± 0.44^b^	78.00 ± 0.42^a^	31.00 ± 0.46^b^	<0.0001
Looks appetizing	44.00 ± 0.50^ab^	61.00 ± 0.49^a^	30.00 ± 0.46^b^	30.00 ± 0.46^b^	<0.0001
Looks healthy	34.00 ± 0.48^a^	27.00 ± 0.45^a^	24.00 ± 0.43^a^	22.00 ± 0.42^a^	0.121
Looks unhealthy	5.00 ± 0.22^a^	6.00 ± 0.24^a^	7.00 ± 0.26^a^	15.00 ± 0.36^a^	0.014
Chocolate aroma	67.00 ± 0.47^a^	71.00 ± 0.46^a^	46.00 ± 0.50^b^	48.00 ± 0.50^b^	<0.0001
Sweet aroma	24.00 ± 0.43^a^	25.00 ± 0.44^a^	21.00 ± 0.41^a^	19.00 ± 0.39^a^	0.613
Aroma (rice, egg, whey)	7.00 ± 0.26^c^	9.00 ± 0.29^bc^	23.00 ± 0.42^ab^	40.00 ± 0.49^a^	<0.0001
Homogeneous appearance	49.00 ± 0.50^ab^	67.00 ± 0.47^a^	40.00 ± 0.49^b^	18.00 ± 0.39^c^	<0.0001
Intense brown color	58.00 ± 0.50^b^	75.00 ± 0.44^a^	13.00 ± 0.34^c^	85.00 ± 0.36^a^	<0.0001
Light brown color	19.00 ± 0.39^b^	7.00 ± 0.26^bc^	64.00 ± 0.48^a^	3.00 ± 0.17^c^	<0.0001

Abbreviations: BWB = buffalo whey beverage; BCC = beverage with calcium caseinate; BPR = beverage with isolated protein rice; BEA = beverage with egg albumin

*Note*: Different lowercase letters on the same line indicate statistically difference (*p* < 0.05) between treatments.

In the BPR treatment, the cocoa flavor characteristic was noted less frequently (*p <* 0.05), and consumers translated it as a product with a “strange taste” that “resembles rice.” Protein products made with soy Protein or other plant‐based proteins were less appealing than dairy products were (Liu et al. 2021). Despite the easy identification of cocoa flavor, the residual characteristic of the egg was also frequently perceived by consumers as “it resembles egg” (36%) and “aroma of Egg” (40%) in the BEA treatment. BWB was categorized as “easy to drink” for 54% of consumers because of the “liquid consistency” noted exclusively in this formulation (*p <* 0.05). BCC was considered a “thick/ creamy” product for almost all volunteers (93%), whereas BPR was perceived most frequently as “sandiness” as the main characteristic.

All products were considered moderately less sweet and had less milk chocolate flavor than ideal, with JAR scores of 2.28 to 2.75 and 2.35 to 2.69, respectively (Table 5). Considering that the proposal for a product rich in protein and fiber attracts an audience more inclined toward healthy foods, positive marketing focusing on health benefits from consuming these products can positively impact the intention to purchase (Tables 2, 6).

**TABLE 5:** JAR profile scores for the different formulations evaluated.

Parameters	BWB	BCC	BPR	BEA	*p‐*Value
Cocoa aroma	3.07 ± 0.94^a^	3.00 ± 0.84^a^	2.88 ± 1.06^a^	2.88 ± 0.97^a^	0.332
Whey aroma	2.86 ± 0.81^a^	2.97 ± 0.74^a^	2.87 ± 1.02^a^	3.04 ± 0.88^a^	0.277
Sweet taste	2.46 ± 0.93^bc^	2.75 ± 0.79^ab^	2.28 ± 0.98^bc^	2.49 ± 0.90^b^	0.001
Acid taste	3.05 ± 0.72^a^	3.07 ± 0.71^a^	3.04 ± 1.03^a^	3.04 ± 0.81^a^	0.988
Light brown color	2.78 ± 0.69^bc^	2.73 ± 0.73^cd^	3.18 ± 0.78^a^	2.50 ± 0.77^d^	<0.0001
Dark brown color	3.08 ± 0.54^b^	3.28 ± 0.72^ab^	2.54 ± 0.71^c^	3.37 ± 0.80^a^	<0.0001
Dark chocolate flavour	3.25 ± 0.98^a^	3.32 ± 0.82^a^	3.09 ± 1.15^a^	3.02 ± 0.96^a^	0.073
Sandy	3.06 ± 0.89^c^	3.27 ± 0.86^bc^	4.18 ± 1.09^a^	3.37 ± 0.86^b^	<0.0001
Consistency	2.43 ± 0.91^b^	3.33 ± 0.95^a^	3.04 ± 0.96^a^	3.06 ± 0.83^a^	<0.0001
Viscosity	2.60 ± 0.87^b^	3.25 ± 0.96^a^	3.06 ± 0.98^a^	3.22 ± 0.79^a^	<0.0001
Mouthfeel	2.52 ± 0.88^c^	3.00 ± 0.98^b^	4.19 ± 1.27^a^	2.70 ± 0.89^bc^	<0.0001

Abbreviations: BWB = buffalo whey beverage; BCC = beverage with calcium caseinate; BPR = beverage with isolated protein rice; BEA = beverage with egg albumin.

*Note*: JAR attributes were evaluated on a 5‐point scale where 3 are considered more proximal to ideal;

Lowercase letters for the same attributes indicate significant differences between treatments (*p <* 0.05).

**TABLE 6:** Consumer penalty analysis of the JAR diagnostic attributes, percentage of consumers (%), and mean drop.

	Flavor and taste							
Formulation	Sweet taste		Acid taste		Milk chocolate flavor		Dark chocolate flavor	
	Too Much	Too little	Too Much	Too little	Too much	Too little	Too much	Too little
BWB	–*	51.0%^§^ (1.09) ^$^	20.0% (1.44)	—	—	48.0% (0.82)	36.0% (1.25)	—
BCC	—	33.0% (2.23)	—	—	—	40.0% (1.57)	30.0% (1.14)	—
BPR	—	60.0% (1.90)	28.0% (1.79)	—	—	56.0% (1.76)	—	30.0% (1.68)
BEA	—	44.0% (1.60)	—	—	—	50.0% (1.47)	—	24.0% (1.47)

	Aroma and appearance							
Formulation	Aroma cocoa		Aroma whey		Light brown		Intense brown	
	Too much	Too little	Too much	Too little	Too much	Too little	Too much	Too little
BWB	‐^–^	25.0% (1.77)	—	27.0% (2.03)	—	—	—	—
BCC	—	21.0% (1.14)	—	—	—	—	—	—
BPR	—	34.0% (1.55)	—	31.0% (1.26)	—	—	—	—
BEA	—	33.0% (1.82)	—	25.0% (2.93)	—	—	—	—

	Texture							
Formulation	Viscosity		Consistency		Sandiness		Mouthfeel	
	Too much	Too little	Too much	Too little	Too much	Too little	Too much	Too little
BWB	—	42.0% (1.59)	—	45.0% (1.30)	–‐	—	—	49.0% (1.77)
BCC	33.0% (0.97)	—	38.0% (1.22)	—	—	—	—	—
BPR	—	—	—	25.0% (1.88)	31.0% (1.04)	—	69.0% (2.73)	—
BEA	—	—	—	—	—	—	—	37.0% (2.32)

Abbreviations: BWB = buffalo whey beverage; BCC = beverage with calcium caseinate; BPR = beverage with isolated protein rice; BEA = beverage with egg albumin.

*Note*: *(‐) indicates that less than 20% of consumers chose this JAR category, or no significant (*p >* 0.05) impact on overall acceptance was observed.

^§^ The percentage of consumers who considered the sensory attribute from JAR scores insufficient or excessive.

^$^ The number in parentheses represents the mean drop, indicating how much the acceptance score dropped due to the nonideal attribute.

Despite the increased consistency and viscosity of the samples subjected to the BCC treatment, they maintained scores that were close to the ideal scores for consumers in general. Only for the BPR treatment was the JAR score considered moderately sandier than ideal, resulting in an unpleasant sensation in the mouth.

In the penalty analysis, all the beverages were made with 50% chocolate (semisweet) and low sugar content (4.0%) and, as a result, were penalized in the “sweet taste” criteria as “too little” for all the treatments (Table 5). Sweet flavor can be considered a predominant sensory attribute that makes food desirable to consumers, and it is a desirable attribute of protein beverages to many consumers (Liu et al. 2021). This reduced percipience in sweet flavor reflects the chocolate flavor criterion, where owing to the presentation of a product with a more intense chocolate flavor than the beverages sold on the market, it ended up creating a false expectation for the consumer's taste, where all the treatments were penalized as having a “too little” chocolate milk flavor (milder chocolate) and, more intensively, BWB and BCC were considered “too much” for the dark chocolate flavor criteria.

Inulin, known for its dietary fiber profile, has emulsifying qualities (mainly as a fat substitute) and has a sweet taste and mild sweetening power (Teferra 2021). Despite the presence of 2.5% inulin in the formulation, the semisweet characteristic of chocolate was predominant, causing penalties in the attribute, mainly due to the consumer's expectation that, because it is a chocolate‐flavored food, a lovely product such as milk chocolate flavor is considered “too little.” However, excessive sugar consumption can contribute to health problems and has been reduced or replaced by healthier sweetening agents such as xylitol (Salgado et al., 2023). Furthermore, health claims, such as high fiber and protein contents or a distinctive appeal to a dark chocolate flavor, can entice consumers to purchase.

With respect to texture, 42.0% to 45.0% of consumers penalized BWB for presenting “too little” viscosity and consistency, and despite 33.0% and 38.0%, respectively, indicating that the increased viscosity and consistency of BCC, the overall impact of linking was lower than that of BWB. BPR has already been penalized for showing little consistency and is the only one with “too Much” sandiness, impairing the mouthfeel criteria (mean drop = 2.73). Mouthfeel is an important attribute that influences the sensation of a product when it is in the mouth, which is related to graininess (sandy texture) (Romulo and Aurellia, 2024). Although chemical and biochemical techniques have advanced to increase the solubility of rice protein, their low solubility is still a limiting factor because extensive aggregation and crosslinking through hydrophobic interactions and disulfide bonds result in insoluble precipitates (Jayaprakash et al. 2022). Considering the results obtained for composition and viscosity, a viable solution to optimize the formulations would be to reduce the amount of protein added while maintaining a content close to 6 g per 100 g and/or mixing protein sources to achieve the best sensory parameters while proposing a more complex and complete protein profile in the products developed.

## 4 Conclusion

The buffalo cocoa beverages presented technological feasibility and microbiological stability, demonstrating their potential for commercialization. Among the formulations, the beverage containing calcium caseinate (BCC) showed the optimal attributes, combining higher viscosity, physicochemical stability, and satisfactory sensory acceptance. At the same time, the control (BWB) and egg albumin (BEA) formulations also achieved good consumer acceptance. In contrast, the rice protein formulation (BPR) was limited by a textural disadvantage, particularly sandiness and grainy mouth feel, which negatively affected sensory perception.

As a byproduct, buffalo whey has potential as a sustainable raw material for the development of functional dairy beverages enriched with proteins and fibers. However, the type of protein source influenced sensory and physicochemical properties, highlighting the intricate nature of ingredient interactions in optimizing product quality and consumer acceptance. Furthermore, these beverages represent an innovative approach to valuing dairy byproducts, contributing to circular‐economy practices and sustainable food production, and promoting environmental and economic benefits for small‐ and medium‐sized dairy producers.

## Author Contributions

**Bruna Samara dos Santos Rekowsky1**: conceptualization, writing – original draft, methodology, formal analysis, visualization. **Katherine Gutiérrez‐álzate**: conceptualization, methodology, formal analysis, writing – original draft. **Joseane Cardoso Gomes de Alencar**: investigation, methodology, formal analysis. **Bruno Nicolau Paulino**: investigation, methodology, formal analysis. **Marion Pereira da Costa**: conceptualization, investigation, funding acquisition, writing – review and editing, project administration, resources.

## Conflicts of Interest

The authors declare no conflicts of interest.

## References

[jfds70999-bib-0001] Ahern, N. , T. Boeck , A. Ressa , et al. 2025. “Decolourised Barley and Rice Protein Isolate – Enhanced Techno‐Functional and Sensory Properties.” Innovative Food Science and Emerging Technologies 102: 103999. 10.1016/j.ifset.2025.103999.

[jfds70999-bib-0002] Akalin, A. S. , G. Unal , N. Dinkci , and A. A. Hayaloglu . 2012. “Microstructural, Textural, and Sensory Characteristics of Probiotic Yogurts Fortified With Sodium Calcium Caseinate or Whey Protein Concentrate.” Journal of Dairy Science 95, no. 7: 3617–3628. 10.3168/jds.2011-5297. 22720919

[jfds70999-bib-0003] Akgun, A. , F. Yazici , and H. A. Gulec . 2016. “Effect of Reduced Fat Content on the Physicochemical and Microbiological Properties of Buffalo Milk Yoghurt.” LWT 74: 521–527. 10.1016/j.lwt.2016.08.015.

[jfds70999-bib-0004] Alfano, A. , S. D'ambrosio , A. D'agostino , R. Finamore , C. Schiraldi , and D. Cimini . 2021. “Concentrated Buffalo Whey as Substrate for Probiotic Cultures and as Source of Bioactive Ingredients: A Local Circular Economy Approach Towards Reuse of Wastewaters.” Fermentation 7, no. 4: 281. 10.3390/fermentation7040281.

[jfds70999-bib-0005] Ali, A. H. , B. Abu‐Jdayil , A. Al Nabulsi , et al. 2023. “Fermented Camel Milk Influenced by Soy Extract: Apparent Viscosity, Viscoelastic Properties, Thixotropic Behavior, and Biological Activities.” Journal of Dairy Science 106, no. 10: 6671–6687. 10.3168/jds.2023-23294. 37562642

[jfds70999-bib-0006] Almeida, C. C. , T. S. Alvares , M. P. Costa , and C. A. Conte‐Junior . 2016. “Protein and Amino Acid Profiles of Different Whey Protein Supplements.” Journal of Dietary Supplements 13, no. 3: 313–323. 10.3109/19390211.2015.1036187. 26317267

[jfds70999-bib-0007] AOAC International . 2016 Official Methods of Analysis of AOAC International. 20th ed., 2 vols. AOAC International.

[jfds70999-bib-0008] APHA . American Public Health Association. 2015. Compendium of Methods for the Microbiological Examination of Foods, Chapter21. 5th ed. Washington, DC, USA.

[jfds70999-bib-0009] Atallah, A. A. , A. Osman , M. Sitohy , et al. 2021. “Physiological Performance of Rabbits Administered Buffalo Milk Yogurts Enriched With Whey Protein Concentrate, Calcium Caseinate or Spirulina Platensis.” Foods 10, no. 10: 2493. 10.3390/foods10102493. 34681542 PMC8535214

[jfds70999-bib-0010] Ba, T. L. , M. S. Dam , L. L. P. Nguyen , L. Baranyai , and T. Kaszab . 2025. “A Review of Processing Techniques and Rheological Properties of Yogurts.” Journal of Texture Studies 56, no. 1: e70006. 10.1111/jtxs.70006. 39909732 PMC11798767

[jfds70999-bib-0011] Barišić, V. , N. C. Icyer , S. Akyil , O. S. Toker , I. Flanjak , and Đ. Ačkar . 2023. “Cocoa Based Beverages – Composition, Nutritional Value, Processing, Quality Problems and New Perspectives.” Trends in Food Science and Technology 132: 65–75. 10.1016/j.tifs.2022.12.011.

[jfds70999-bib-0012] Brazil. 2018. Organizaçao das Naçoes Unidas no Brasil. Objetivos de Desenvolvimento Sustentável [Internet]. Disponível em:. https://brasil.un.org/pt‐br/sdgs.

[jfds70999-bib-0013] Brazil. 2020 Instrução Normativa N° 75, de 8 de outubro de 2020. Estabelece os requisitos técnicos para a declaração da rotulagem nutricional dos alimentos embalados, Diario Oficial da Uniao, Brasília, Brasil.

[jfds70999-bib-0014] Cacciola, N. A. , A. Salzano , N. D'Onofrio , et al. 2022. “Buffalo Milk Whey Activates Necroptosis and Apoptosis in a Xenograft Model of Colorectal Cancer.” International Journal of Molecular Sciences 23, no. 15: 8464. 10.3390/ijms23158464. 35955595 PMC9368892

[jfds70999-bib-0015] Chalupa‐Krebzdak, S. , C. J. Long , and B. M. Bohrer . 2018. “Nutrient Density and Nutritional Value of Milk And Plant‐Based Milk Alternatives.” International Dairy Journal 87: 84–92. 10.1016/j.idairyj.2018.07.018.

[jfds70999-bib-0016] Cheng, N. , D. M. Barbano , and M. A. Drake . 2018. “Effect of Pasteurization and Fat, Protein, Casein to Serum Protein Ratio, and Milk Temperature on Milk Beverage Color and Viscosity.” Journal of Dairy Science 102, no. 3: 2022–2043. 10.3168/jds.2018-15739 30612790

[jfds70999-bib-0017] Di Paolo, M. , V. Pelizzola , L. De Luca , et al. 2025. “Effect of Technological Process and Temperature on Phospholipids in Buffalo Milk, Whey and Buttermilk.” Foods 14, no. 15: 2756. 10.3390/foods14152756. 40807692 PMC12346082

[jfds70999-bib-0018] Guimarães, J. T. , E. K. Silva , A. L. R. Costa , et al. 2018. “Manufacturing a Prebiotic Whey Beverage Exploring the Influence of Degree of Inulin Polymerization.” Food Hydrocolloids 77: 787–795. 10.1016/j.foodhyd.2017.11.021.

[jfds70999-bib-0019] Gupta, H. R. , S. K. Kanawjia , M. K. Salooja , P. Sharma , and A. Kumar . 2017. “Physico‐Chemical and Microbiological Quality Changes in Cocoa and Whey Protein Enriched Functional Dairy Drink During Storage.” In Indian Journal of Dairy Science 70, no. 3.

[jfds70999-bib-0020] Gupta, S. S. , and M. Ghosh . 2015. “Formulation Development and Process Parameter Optimization of Lipid Nanoemulsions Using an Alginate‐Protein Stabilizer.” Journal of Food Science and Technology 52: 2544–2557. 10.1007/s13197-014-1348-0. 25892754 PMC4397346

[jfds70999-bib-0073] Gutiérrez‐Álzate, K. , G. B. Abreu , B. S. S. Rekowsky , L. P. R. Alencar , D. M. Otero , and M. P. Costa . 2025. “Microencapsulation of Pediococcus pentosaceus using cupuassu flour‐based as wall material: Application and impact on petit suisse.” Food bioscience 7: 107071. 10.1016/j.fbio.2025.107071.

[jfds70999-bib-0021] He, C. , Y. Hu , Z. Liu , M. W. Woo , H. Xiong , and Q. Zhao . 2020. “Interaction Between Casein and Rice Glutelin: Binding Mechanisms and Molecular Assembly Behaviours.” Food Hydrocolloids 107: 105967. 10.1016/j.foodhyd.2020.105967.

[jfds70999-bib-0022] Helal, Y. M. , S. A. Awad , A. M. Naem , M. Y. El‐Hawary , and A. S. Bakr . 2023. “Utilization of Egg White in the Production of High‐Protein Milk Beverages.” Journal of Sustainable Agricultural and Environmental Sciences 2, no. 2: 56–65.

[jfds70999-bib-0023] Holt, C. , and J. A. Carver . 2024. “Invited Review: Modeling milk stability.” Journal of Dairy Science 107, no. 8: 5259–5279. 38522835 10.3168/jds.2024-24779

[jfds70999-bib-0024] Huecker, M. , M. Sarav , M. Pearlman , and J. Laster . 2019. “Protein Supplementation in Sport: Source, Timing, and Intended Benefits.” Current Nutrition Reports 8: 382–396. 10.1007/s13668-019-00293-1. 31713177

[jfds70999-bib-0025] Jang, H. J. , J. H. Kim , H. S. Lee , and H. D. Paik . 2022. “Physicochemical Analysis of Non‐Fermented Probiotic Milk With Probiotic *Lactobacillus Plantarum* Ln1 Isolated From Korea Traditional Fermented Food.” Food Science and Biotechnology 31: 731–737. 10.1007/s10068-022-01076-1. 35646416 PMC9133277

[jfds70999-bib-0026] Jang, H. J. , N. K. Lee , and H. D. Paik . 2024. “Overview of Dairy‐Based Products With Probiotics: Fermented or Non‐Fermented Milk Drink.” Food Science of Animal Resources 44, no. 2: 255–268. 10.5851/KOSFA.2023.E83. 38764505 PMC11097033

[jfds70999-bib-0027] Jayaprakash, G. , A. Bains , P. Chawla , M. Fogarasi , and S. Fogarasi . 2022. “A Narrative Review on Rice Proteins: Current Scenario and Food Industrial Application.” Polymers 14, no. 15: 3003. 10.3390/polym14153003. 35893967 PMC9370113

[jfds70999-bib-0028] Juthi, L. M. , N. Yeasmen , T. Kabir , N. Podder , and M. G. Aziz . 2025. “Separation and Characterization of Whey Protein Powder From Cheese By‐Product.” Applied Food Research 5, no. 2: 10139.

[jfds70999-bib-0029] Juvinal, J. G. , H. De Steur , J. J. Schouteten , et al. 2023. “Physico‐Chemical Property, Sensory Profile and Consumer Acceptability of Water Buffalo (*Bubalus bubalis L*.) Chocolate Milk Using Alkalized and Natural Cocoa Powder.” Foods 12, no. 9: 1797. 10.3390/foods12091797. 37174335 PMC10178308

[jfds70999-bib-0030] Kamel, D. G. , A. R. A. Hammam , K. A. Alsaleem , and D. M. Osman . 2021. “Addition of Inulin to Probiotic Yogurt: Viability of Probiotic Bacteria (*Bifidobacterium Bifidum*) and Sensory Characteristics.” Food Science and Nutrition 9, no. 3: 1743–1749. 10.1002/fsn3.2154. 33747485 PMC7958560

[jfds70999-bib-0031] Kapila, R. , P. K. Kavadi , and S. Kapila . 2013. “Comparative Evaluation of Allergic Sensitization to Milk Proteins of Cow, Buffalo and Goat.” Small Ruminant Research 112, no. 1–3: 191–198. 10.1016/j.smallrumres.2012.11.028.

[jfds70999-bib-0032] Kumar, M. D. , M. Anupama , M. D. Baig , A. K. Beena , and S. N. Rajakumar . 2021. “Development and Characterisation of Synbiotic Whey Beverage.” Indian Journal of Dairy Science 74, no. 3: 208–214.

[jfds70999-bib-0033] Liu, Y. , R. S. D. Toro‐Gipson , and M. A. Drake . 2021. “Sensory Properties and Consumer Acceptance Of Ready‐to‐Drink Vanilla Protein Beverages.” Journal of Sensory Studies 36, no. 6: e12704. 10.1111/joss.12704.

[jfds70999-bib-0034] López‐Castejón, M. L. , C. Bengoechea , S. Espinosa , and C. Carrera . 2019. “Characterization of Prebiotic Emulsions Stabilized by Inulin and Β‐Lactoglobulin.” Food Hydrocolloids 87: 382–393. 10.1016/j.foodhyd.2018.08.024.

[jfds70999-bib-0035] Lotfian, F. , Z. Emam Djomeh , M. Karami , and S. Moeini . 2019. “Protein Beverages Made of a Mixture of Egg White and Chocolate Milk: Microbiology, Nutritional and Sensory Properties.” Food Science and Nutrition 7, no. 4: 1466–1472. 10.1002/fsn3.983. 31024720 PMC6475758

[jfds70999-bib-0036] Magalhães, K. T. , D. R. Dias , G. V. de Melo Pereira , et al. 2011. “Chemical Composition and Sensory Analysis of Cheese Whey‐Based Beverages Using Kefir Grains as Starter Culture.” International Journal of Food Science and Technology 46, no. 4: 871–878. 10.1111/j.1365-2621.2011.02570.x.

[jfds70999-bib-0037] Manassero, C. A. , M. C. Añón , and F. Speroni . 2020. “Development of a High Protein Beverage Based on Amaranth.” Plant Foods for Human Nutrition 75: 599–607. 10.1007/s11130-020-00853-9. 32939740

[jfds70999-bib-0038] Mehta, D. , M. H. S. Kumar , and L. Sabikhi . 2017. “Development of High Protein, High Fiber Smoothie as a Grab‐and‐go Breakfast Option Using Response Surface Methodology.” Journal of Food Science and Technology 54: 3859–3866. 10.1007/s13197-017-2841-z. 29085128 PMC5643802

[jfds70999-bib-0039] Montero‐Zamora, J. , M. Cortés‐Muñoz , P. Esquivel , J. A. Mora‐Villalobos , and C. Velázquez . 2020. “Growth Conditions and Survival Kinetics During Storage of *Lactobacillus Rhamnosus* GG for the Design of a Sustainable Probiotic Whey‐Based Beverage Containing Costa Rican Guava Fruit Pulp.” Journal of Food Science 85, no. 10: 3478–3486. 10.1111/1750-3841.15430. 32901935

[jfds70999-bib-0040] Morais, R. , P. I. Soares , S. K. Morais , et al. 2023. “Development and Characterization of Symbiotic Buffalo Petit Suisse Cheese Utilizing Whey Retention and Inulin Incorporation.” Foods 12, no. 23: 4343. 10.3390/foods12234343. 38231859 PMC10705983

[jfds70999-bib-0041] Ostertag, F. , C. M. Schmidt , S. Berensmeier , and J. Hinrichs . 2021. “Development and Validation of an RP‐HPLC DAD Method for the Simultaneous Quantification of Minor and Major Whey Proteins.” Food Chemistry 342: 128176. 10.1016/j.foodchem.2020.128176. 33046286

[jfds70999-bib-0042] Otero, D. M. , G. d. R. L. Mendes , A. J. da Silva Lucas , A. Christ‐Ribeiro , and C. D. F. Ribeiro . 2022. “Exploring Alternative Protein Sources: Evidence From Patents and Articles Focusing on Food Markets.” Food Chemistry 394: 133486. 10.1016/j.foodchem.2022.133486. 35759839

[jfds70999-bib-0043] Panda, P. , S. Meena , K. Meena , D. C. Rai , D. S. Bunkar , and P. B. Gautam . 2023. “Development of Functional Milk‐Based Smoothie by Incorporating Horse Gram Extract.” Current Research in Nutrition and Food Science 11, no. 3. 10.12944/CRNFSJ.11.3.26.

[jfds70999-bib-0044] Panghal, A. , V. Kumar , S. B. Dhull , Y. Gat , and N. Chhikara . 2017. “Utilization of Dairy Waste‐Whey in Formulation of Papaya RTS Beverage.” Current Research in Nutrition and Food Science 5, no. 2: 168–174.

[jfds70999-bib-0045] Parhi, P. , K. P. Song , and W. S. Choo . 2021. “Viability, Storage Stabilityand in Vitro Gastrointestinal Tolerance of *Lactiplantibacillus Plantarum* Grown in Model Sugar Systems With Inulin and Fructooligosaccharide Supplementation.” Fermentation 7, no. 4: 259. 10.3390/fermentation7040259.

[jfds70999-bib-0046] Pérez‐Ramírez, I. F. , A. Cariñ O‐Sarabia , E. Castañ O‐Tostado , et al. 2021. “Chemical and Sensorial Characterization of Tejate, A Mexican Traditional Maize‐Cocoa Beverage, and Improvement of its Nutritional Value by Protein Addition.” Journal of Food Science and Technology 58: 3548–3560. 10.1007/s13197. 34366472 PMC8292523

[jfds70999-bib-0047] Puglisi, M. J. , and M. L. Fernandez . 2022. “The Health Benefits of Egg Protein.” Nutrients 14, no. 14: 2904. 10.3390/nu14142904. 35889862 PMC9316657

[jfds70999-bib-0048] Queiroz, C. S. , B. S. S. Rekowsky , M. Johel , et al. 2025. Development of Fermented Milks With *Lacticaseibacillus Casei B5* and *Lactiplantibacillus Plantarum B7* Isolated from Minas Artisanal Cheese. Fermentation 11, no. 10. 10.3390/fermentation11100560.

[jfds70999-bib-0049] Rafiq, S. , N. Huma , I. Pasha , A. Sameen , O. Mukhtar , and M. I. Khan . 2016. “Chemical Composition, Nitrogen Fractions and Amino Acids Profile of Milk From Different Animal Species.” Asian‐Australasian Journal of Animal Sciences 29, no. 7: 1022–1028. 10.5713/ajas.15.0452. 26954163 PMC4932579

[jfds70999-bib-0050] Rani, R. , L. Sabikhi , and M. H. Sathish Kumar . 2024. “Storage Stability, Nutritional Profiling and Consumer Acceptability of a Milk‐Sorghum‐Based Breakfast Smoothie.” Sustainable Food Technology 2, no. 3: 729–740. 10.1039/d4fb00038b.

[jfds70999-bib-0051] Rashid, A. A. , S. Saeed , I. Ahmad , K. Shehzad , S. Nawaz , and S. Inayat . 2023. “Development of Ricotta Cheese From Concentrated Buffalo Cheese Whey by Optimization of Processing Conditions Using RSM.” Journal of Food Measurement and Characterization 17: 4739–4746. 10.1007/s11694-023-02004-5.

[jfds70999-bib-0052] Rekowsky, B. S. S. , M. L. G. Monteiro , T. M. Silva , C. A. Conté‐Júnior , and M. P. Da Costa . 2022. “Semi‐Hard Buffalo Cheese: How Cow's Milk Affects Sensory Acceptance?.” Brazilian Journal of Food Technology 25: 00. 10.1590/1981-6723.03022.

[jfds70999-bib-0053] Remeuf, F. , S. Mohammed , I. Sodini , and J. P. Tissier . 2003. “Preliminary Observations on the Effects of Milk Fortification and Heating on Microstructure and Physical Properties Of Stirred Yogurt.” International Dairy Journal 13, no. 9: 773–782. 10.1016/S0958-6946(03)00092-X.

[jfds70999-bib-0054] Romulo, A. , and C. A. Aurellia . 2024. “Different Concentrations of Maltodextrin and Albumin Influenced the Quality Characteristics and Hedonic Acceptance of Sorghum Powder Drinks.” *IOP Conference Series: Earth Enviromental Science* 1338: 012029.

[jfds70999-bib-0055] Rosa, M. C. , M. R. S. Carmo , C. F. Balthazar , et al. 2021. “Dairy Products With Prebiotics: An Overview of the Health Benefits, Technological and Sensory Properties.” International Dairy Journal 117: 105009. 10.1016/j.idairyj.2021.105009.

[jfds70999-bib-0057] Sales, D. C. , A. H. d. N. Rangel , S. A. Urbano , et al. 2018. “Buffalo Milk Composition, Processing Factors, Whey Constituents Recovery and Yield in Manufacturing Mozzarella Cheese.” Food Science and Technology 38, no. 2. 10.1590/1678-457X.04317.

[jfds70999-bib-0058] Salgado, M. J. G. , I. L. S. Rosario , A. C. de Oliveira Almeida , et al. 2023. “Buffalo Whey‐Based Cocoa Beverages With Unconventional Plant‐Based Flours: The Effect of Information and Taste on Consumer Perception.” Beverages 9, no. 4: 90. 10.3390/beverages9040090.

[jfds70999-bib-0059] Silva, T. M. S. , A. C. M. Piazentin , C. M. N. Mendonça , et al. 2020. “Buffalo Milk Increases Viability And Resistance of Probiotic Bacteria in Dairy Beverages Under in Vitro Simulated Gastrointestinal Conditions.” Journal of Dairy Science 103, no. 9: 7890–7897. 10.3168/jds.2019-18078. 32600759

[jfds70999-bib-0060] Singh, H. , and A. Ye . 2019. “Interactions and Functionality of Milk Proteins in Food Emulsions.” In Milk Proteins: From Expression to Food: 467–497. 10.1016/B978-0-12-815251-5.00012-8.

[jfds70999-bib-0061] Sobti, B. , M. Mbye , H. Alketbi , et al. 2020. “Rheological Characteristics and Consumer Acceptance of Camel Milk Yogurts as Affected by Bovine Proteins and Hydrocolloids.” International Journal of Food Properties 23, no. 1: 1347–1360. 10.1080/10942912.2020.1797785.

[jfds70999-bib-0062] Teferra, T. F. 2021. “Possible Actions of Inulin as Prebiotic Polysaccharide: A Review.” Food Frontiers 2, no. 4: 407–416. 10.1002/fft2.92.

[jfds70999-bib-0063] Thomar, P. , T. Nicolai , L. Benyahia , and D. Durand . 2013. “Comparative Study of the Rheology and Structure of Sodium and Calcium Caseinate Solutions.” International Dairy Journal 31, no. 2: 100–106.

[jfds70999-bib-0064] Torres, I. C. , J. M. Amigo , J. C. Knudsen , A. Tolkach , B. Ø. Mikkelsen , and R. Ipsen . 2018. “Rheology and Microstructure of Low‐Fat Yoghurt Produced With Whey Protein Microparticles as Fat Replacer.” International Dairy Journal 81: 62–71. 10.1016/j.idairyj.2018.01.004.

[jfds70999-bib-0065] UN . 2015. United Nations The 17 Sustainable Development Goals (SDGs).. Available at: https://sdgs.un.org/goals. Accessed on: 11/05/2025.

[jfds70999-bib-0066] Vasquez‐Orejarena, E. , C. T. Simons , J. H. Litchfield , and V. B. Alvarez . 2018. “Functional Properties of a High Protein Beverage Stabilized With Oat‐β‐Glucan.” Journal of Food Science 83, no. 5: 1360–1365. 10.1111/1750-3841.14119. 29603228

[jfds70999-bib-0067] Wang, R. , P. Xu , Z. Chen , X. Zhou , and T. Wang . 2019. “Complexation of Rice Proteins and Whey Protein Isolates by Structural Interactions to Prepare Soluble Protein Composites.” LWT 101: 207–213. 10.1016/j.lwt.2018.11.006.

[jfds70999-bib-0068] Wang, T. , F. Liu , R. Wang , L. Wang , H. Zhang , and Z. Chen . 2015. “Solubilization by Freeze‐Milling of Water‐Insoluble Subunits in Rice Proteins.” Food and Function 6, no. 2: 423–430. 10.1039/c4fo00828f. 25412155

[jfds70999-bib-0069] Wang, T. , P. Xu , Z. Chen , X. Zhou , and R. Wang . 2018. “Alteration of the Structure of Rice Proteins by Their Interaction With Soy Protein Isolates to Design Novel Protein Composites.” Food and Function 9, no. 8: 4282–4291. 10.1039/c8fo00661j. 30033456

[jfds70999-bib-0070] Yang, J. , D. Meng , Z. Wu , J. Chen , and L. Xue . 2023. “Modification and Solubility Enhancement of Rice Protein and its Application in Food Processing: A Review.” Molecules 28, no. 10: 4078. 10.3390/molecules28104078. 37241820 PMC10223372

[jfds70999-bib-0071] Zaman, Q. U. , A. Sahar , A. Sameen , et al. 2023. “Development and Storage Stability of Whey Sugarcane Based Functional Beverage.” International Journal of Food Properties 26, no. 1: 752–763. 10.1080/10942912.2023.2183170.

[jfds70999-bib-0072] Zhang, Y. , D. Li , Y. Diao , et al. 2024. “Effect of Rice Bran Protein on the Foaming Properties and Foaming Characteristics of Rice Bran Protein–Sodium Caseinate and Rice Bran Protein Nanoparticles–Sodium Caseinate.” Foods 13, no. 15: 2328. 10.3390/foods13152328. 39123519 PMC11311429

